# Regulation of glucose metabolism in T cells: new insight into the role of Phosphoinositide 3-kinases

**DOI:** 10.3389/fimmu.2012.00247

**Published:** 2012-08-07

**Authors:** David K. Finlay

**Affiliations:** ^1^School of Biochemistry and Immunology, Trinity Biomedical Sciences Institute, Trinity College Dublin,Dublin, Ireland; ^2^School of Pharmacy and Pharmaceutical Sciences, Trinity Biomedical Sciences Institute, Trinity College Dublin,Dublin, Ireland

**Keywords:** PI3K, Glucose metabolism, Akt, T lymphocyte, aerobic glycolysis, c-Myc, PDK1

## Abstract

Naïve T cells are relatively quiescent cells that only require energy to prevent atrophy and for survival and migration. However, in response to developmental or extrinsic cues T cells can engage in rapid growth and robust proliferation, produce of a range of effector molecules and migrate through peripheral tissues. To meet the significantly increased metabolic demands of these activities, T cells switch from primarily metabolizing glucose to carbon dioxide through oxidative phosphorylation to utilizing glycolysis to convert glucose to lactate (termed aerobic glycolysis). This metabolic switch allows glucose to be used as a source of carbon to generate biosynthetic precursors for the production of protein, DNA, and phospholipids, and is crucial for T cells to meet metabolic demands. Phosphoinositide 3-kinases (PI3K) are a family of inositol lipid kinases linked with a broad range of cellular functions in T lymphocytes that include cell growth, proliferation, metabolism, differentiation, survival, and migration. Initial research described a critical role for PI3K signaling through Akt (also called protein kinase B) for the increased glucose uptake and glycolysis that accompanies T cell activation. This review article relates this original research with more recent data and discusses the evidence for and against a role for PI3K in regulating the metabolic switch to aerobic glycolysis in T cells.

## PI3K IN T CELLS

**FIGURE 1:**
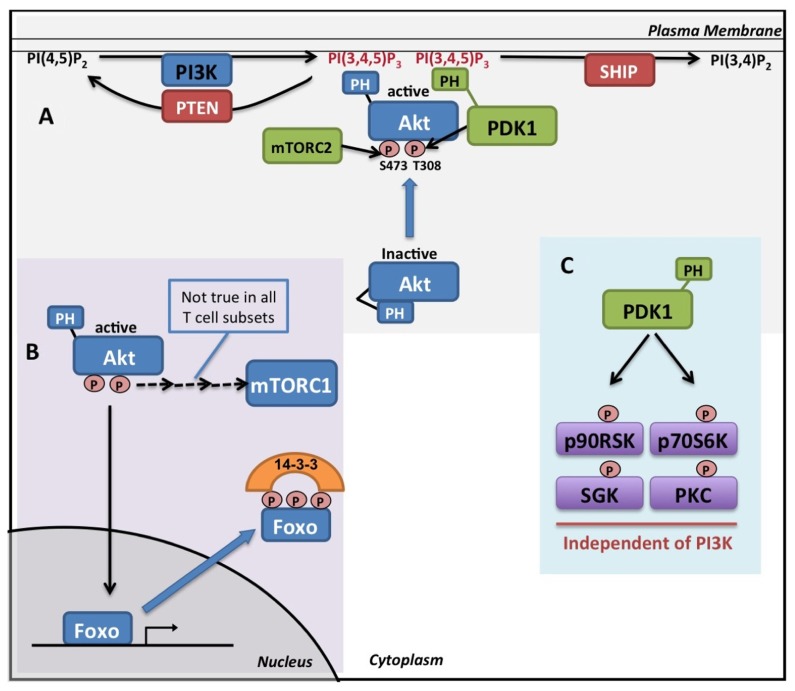
**Pl3K and Akt signaling.** **(A)** PI(4,5)P_2_ is phosphorylated by Class 1 PI3K isoforms generating the second messenger molecule PI(3,4,5)P_3_. Levels of PI(3,4,5)P_3 _ are negatively regulated by the lipid phosphatases PTEN and SHIP. Binding of the PH domain of Akt to PI(3,4,5)P_3_ in the membrane results in a conformational change that allows for the phosphorylation of Akt on key residues(T308 and S473) by PDK1 and mTORC2. The recruitment of PDK1, via its PH domain, to the site of PI(3,4,5)P_3 _ is required for efficient Akt activation. **(B) **Active Akt phosphorylates Foxo transcription factors on multiple sites resulting in their translocation to the cytoplasm where they are retained in complex with 14-3-3 proteins. mTORC1 can be activated downstream of Akt, though this does not occur in all T cell types. **(C) **PDK1 also phosphorylates and activates a number of other members of the AGC kinase family in a PI3K independent manner.

Class 1 phosphoinositide 3-kinases (PI3K), lipid kinases that phosphorylate phosphatidylinositol-(4,5)-bisphosphate [PI(4,5)P_2_] to generate the lipid signaling molecule phosphatidylinositol-(3,4,5)-trisphosphate [PI(3,4,5)P_3_], play a crucial role in many aspects of T cell biology (Okkenhaug and Vanhaesebroeck, 2003). Class 1 PI3Ks consist of a catalytic subunit, responsible for the lipid kinase activity, and an adapter subunit which links the catalytic subunit to upstream activating signals. They are subdivided into Class 1A; p110α, β, or δ catalytic subunits coupled to p85 adapter subunits, and Class 1B; p110γ catalytic subunit coupled to the p101 adapter protein. The p85 subunit couples Class 1A PI3Ks to docking sites created by tyrosine-kinase signaling while the Class IB p101 links the p110γ kinase with G-protein-coupled receptors (Vanhaesebroeck et al., 2010). The levels of the lipid signaling molecule PI(3,4,5)P_3_ are coordinately regulated by both class 1 PI3Ks and by the action of 3′ and 5′ phosphatases PTEN and SHIP1 that dephosphorylate PI(3,4,5)P_3_to generate PI(4,5)P_2_ and PI(3,4)P_2_ respectively (**Figure 1A**).

PI(3,4,5)P_3_ is an important lipid signaling molecule in T cells, present at low levels in naïve T cells and elevated in response to signaling initiated by the T cell receptor (TCR) and various cytokine and chemokine receptors (Koyasu, 2003). Following TCR engagement by antigen presenting cells (APC) levels of PI(3,4,5)P_3_ accumulate in the plasma membrane and are maintained for prolonged periods, requiring continual TCR engagement and activation of PI3K (Costello et al., 2002; Harriague and Bismuth, 2002; Huppa et al., 2003). Signals from co-stimulatory molecules such as CD28 are important for sustaining PI(3,4,5)P_3_ levels following TCR engagement but are not in themselves sufficient to stimulate PI3K as PI(3,4,5)P_3_ levels are only induced when the TCR engages APC presenting cognate peptide antigen (Costello et al., 2002; Garcon et al., 2007). Other physiological stimuli that can stimulate PI(3,4,5)P_3_ levels in T cells include cytokines and chemokines (Koyasu, 2003). While PI(3,4,5)P_3_ levels are induced by a number of cytokines it should be noted that the potency of these different cytokines can vary, e.g., IL2 maintains high levels of PI(3,4,5)P_3_ while IL15 maintains comparatively low levels (Cornish et al., 2006; Sinclair et al., 2008).

In the thymus, PI3Kδ and PI3Kγ are the major isoforms required in developing thymocytes. While deletion of PI3Kδ or γ individually does not have a pronounced effect on thymopoiesis, deletion or inactivation of both isoforms in developing T cells results in a block early in thymocyte development at the CD4 CD8 double negative (DN) stage (Sasaki et al., 2000; Okkenhaug et al., 2002; Webb et al., 2005; Swat et al., 2006; Ji et al., 2007). Negative regulation of PI(3,4,5)P_3_ signaling is also important in developing thymocytes. Thus, deletion of PTEN in early thymocyte progenitors results in constitutive PI(3,4,5)P_3_ signaling that leads to the development of T cell leukemia or lymphoma (Suzuki et al., 2001; Hagenbeek and Spits, 2008; Finlay et al., 2009). In contrast to developing thymocytes, in mature T cells PI3Kδ appears to be the major PI3K isoform responsible for promoting PI(3,4,5)P_3_ signaling in activated T cells. Thus, a point mutation that makes p110δ catalytically inactive (D910A) abolishes PI(3,4,5)P_3_ signaling in activated T cell subsets (Okkenhaug et al., 2002, 2006; Macintyre et al., 2011).

## PI3K SIGNALING

PI(3,4,5)P_3_ acts as a signaling molecule through its interaction with the pleckstrin homology (PH) domains of a diverse array of signal transduction proteins. These proteins include Akt (also called PKB), Tec family kinases, and guanine-nucleotide-exchange proteins for Rho family GTPases. This interaction primarily controls the subcellular localization of PH domain containing proteins but can also control protein conformation and enzyme activity. Consider, for example, the activation of Akt, which is the best characterized PI(3,4,5)P_3_ effector in T cells (for reviews Alessi and Cohen, 1998; Hanada et al., 2004; Cameron et al., 2007). The interaction of PI(3,4,5)P_3_ with the PH domain of Akt stimulates its kinase activity by inducing a conformational change that allows Akt to be phosphorylated on threonine 308 and serine 473 by its upstream activating kinases phosphoinositide-dependent kinase 1 (PDK1) and mechanistic Target Of Rapamycin Complex 2 (mTORC2) respectively (**Figure 1A;** Calleja et al., 2007). PDK1 also contains a PH domain and co-localization of Akt and PDK1 to sites of PI(3,4,5)P_3_ is required for efficient Akt activation (Bayascas et al., 2008; Waugh et al., 2009). Once activated, Akt phosphorylates a number of important signaling molecules including the Foxo transcription factors (Manning and Cantley, 2007). Foxo transcription factors localize to the nucleus where they promote the expression of target genes. Once phosphorylated by Akt, Foxos translocate into the cytoplasm where they are retained through their interaction with 14-3-3 proteins (**Figure1B;** Coffer and Burgering, 2004; Burgering, 2008). PI3K/Akt signaling also activates the mTOR Complex 1 (mTORC1) in many cellular systems through multiple mechanisms (Laplante and Sabatini, 2009). mTORC1 is an important regulator of cellular metabolism that senses environmental cues such as nutrient availability and energy homeostasis (Delgoffe and Powell, 2009). However, it is now becoming apparent that mTORC1 activity is not universally dependent upon PI3K/Akt signaling in T cells. Thus, in activated CD8 cells, mTORC1 activity is not blocked by the disruption of PI3K/Akt signaling by various pharmacological and genetic strategies (unpublished data; Macintyre et al., 2011).

## MATCHING GLUCOSE METABOLISM TO METABOLIC DEMANDS

**FIGURE 2:**
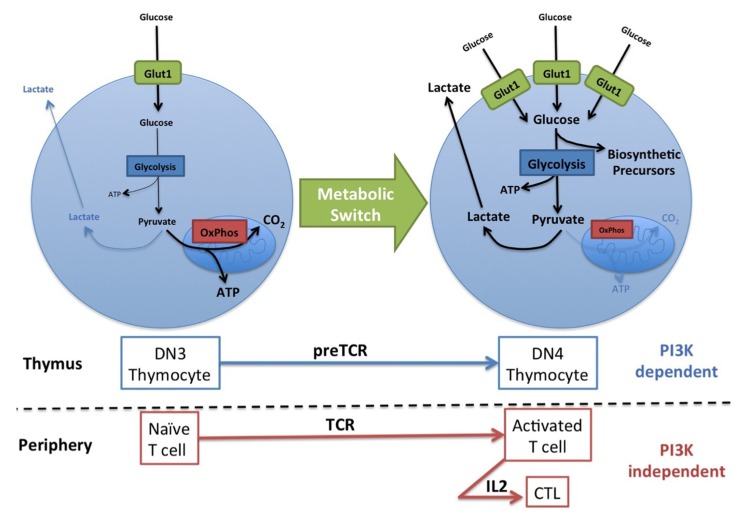
**PI3K controls the metabolic switch to aerobic glycolysis in thymocytes but not in mature T cells.** Naïve T cells take up small amounts of glucose which is for the most part metabolized to CO_2_ via mitochondrial oxidative phosphorylation (OxPhos) for the efficient generation of ATP. Activated T cells and certain thymocyte subsets increase the expression of the GLUT1 glucose transporter and thus glucose uptake, and increase glycolytic flux, primarily converting glucose to lactate. This metabolic switch increases the biosynthetic capacity of the T cells and is stimulated by antigen receptor signaling (PreTCR, TCR) and maintained by certain cytokines (IL2). PI3K signaling is required for this metabolic switch during thymopoiesis but not in peripheral activated T cells.

While naïve T cells only require energy to prevent atrophy and for survival and migration, activated T cell subsets have a greatly increased metabolic demand as they engage in rapid growth and proliferation, and the production of cytokines and other effector molecules. It is crucial that activated T cells increase their metabolism to meet the biosynthetic needs of the T cell as it responds either to developmental or pathogenic cues. To achieve this T cells respond to extrinsic signals from antigen receptors and cytokines to up-regulate the surface expression of key nutrient receptors: amino acid transporters, the transferrin receptor, and glucose transporters (Fox et al., 2005a; Kelly et al., 2007; Jacobs et al., 2008). Additionally, T cells switch their glucose metabolism from oxidative phosphorylation to aerobic glycolysis; i.e., glucose is metabolized to produce lactate even though oxygen is readily available (**Figure 2;** Greiner et al., 1994). Aerobic glycolysis is an inefficient route to generating ATP, producing two molecules ATP per molecule of glucose compared to >30 molecules ATP per glucose generated by oxidative phosphorylation. Therefore, cells must be able to sustain high levels of glucose uptake and an elevated glycolytic flux to generate sufficient ATP. This is achieved by increasing the expression of the GLUT1 glucose transporter and certain rate limiting enzymes within the glycolytic pathway (Vander Heiden et al., 2009; Marko et al., 2010). However, the real advantage of switching from oxidative phosphorylation to glycolysis is that it allows glucose to be used as a source of carbon to generate nucleic acid, amino acids and phospholipids (**Figure 2;** Vander Heiden et al., 2009). The generation of these biosynthetic precursors is critical for cells engaging in rapid growth, proliferation, and the synthesis of effector molecules. Therefore, to facilitate their differentiation and function, activated T cells up-regulate the expression of GLUT1, increase glucose uptake, and activate the switch to aerobic glycolysis (Brand et al., 1984; Greiner et al., 1994; Frauwirth et al., 2002; Wofford et al., 2008).

## PI3K AND GLUCOSE METABOLISM IN THE THYMUS

In the thymus discrete subpopulations, e.g., DN4 thymocytes, engage in rapid growth and robust proliferation. PI3K and Akt signaling is crucial in allowing these thymocyte subsets to match their metabolism with metabolic demands (Ciofani and Zuniga-Pflucker, 2005; Juntilla et al., 2007; Kelly et al., 2007; Finlay et al., 2010). Thus, mice lacking Akt or PDK1 or both PI3Kδ and γ isoforms during early thymopoiesis have a profound developmental block at the DN3/DN4 stage of T cell development(Hinton et al., 2004; Webb et al., 2005; Swat et al., 2006; Fayard et al., 2007; Juntilla et al., 2007; Mao et al., 2007). In the absence of PI3K/PDK1/Akt signaling DN4 thymocytes fail to up-regulate the expression of the glucose transporter, GLUT1, and also the expression of other key nutrient receptors for the uptake of amino acids (CD98, component of the L-amino acid transporter) and iron (transferrin receptor, CD71). Increased provision of these nutrients is a key requirement for these cells to meet the metabolic demands of rapid growth and proliferation and PI3K/PDK1/Akt deficient DN4 thymocytes that fail to do so atrophy and fail to develop (Juntilla et al., 2007; Kelly et al., 2007). Therefore, in T cells developing in the thymus, PI3K and Akt signaling is crucial to allow certain thymocyte subsets to match glucose metabolism with metabolic demands.

## PI3K AND GLUCOSE METABOLISM IN ACTIVATED T CELLS

The metabolic switch to aerobic glycolysis is crucial during the activation and differentiation of T cells in the periphery. Thus, limiting glucose availability in activating T cells compromises TCR induced growth and proliferation and also the expression of certain effector molecules such as interferon γ (IFNγ; Cham et al., 2008; Jacobs et al., 2008). The transcription factor c-Myc is crucial for the metabolic switch in glucose metabolism that accompanies the activation of naïve T cells (Wang et al., 2011). Accordingly, deletion of c-Myc in naïve T cells prevents TCR induced glucose uptake and glycolysis, and activated c-Myc-null T cells completely fail to grow or proliferate (Trumpp et al., 2001; Iritani et al., 2002; Dose et al., 2006; Wang et al., 2011). Is PI3K and Akt signaling also required for the increase in glucose uptake and glycolysis in TCR activated T cells in the periphery? Certainly, antigen receptor induced c-Myc expression and glucose uptake have been attributed to PI3K signaling (Frauwirth et al., 2002; Grumont et al., 2002; Doughty et al., 2006; Jacobs et al., 2008; Wang et al., 2011). However, one criticism of these studies is that they rely on experiments involving the overexpression of Akt and the use of the PI3K inhibitor LY294002. Overexpression studies can be difficult to interrupt and while LY294002 was initially believed to be a highly specific PI3K inhibitor, and as such was used in good faith, it has since emerged that this inhibitor is rather non-specific. LY294002 potently inhibits a number kinases other than PI3K, including those with described roles in regulating T cell growth and proliferation, i.e., mTORC1 and Pim family kinases (Brunn et al., 1996; Davies et al., 2000; Fox et al., 2005b; Bain et al., 2007). The importance of these other LY204002 targets for T cell metabolism can be appreciated by a comparison of the cellular sizes of PI3K/Akt deficient CD8 cytotoxic T lymphocytes (CTL) and wild-type CTL cultured in the presence of LY294002. While PI3K/Akt deficient CTL are comparable in size to wild-type CTL, LY294002-treated CTL are substantially smaller (Cornish et al., 2006; Sinclair et al., 2008; Macintyre et al., 2011). Therefore, the question as to whether PI3K regulates TCR induced c-Myc expression has not been satisfactorily investigated to date. Nevertheless, a comparison of PI3K/Akt and c-Myc deficient T cells is extremely informative. In contrast to the failure of c-Myc-null T cells to engage in TCR stimulated growth and proliferation, PI3K deficient T cells show a relatively mild defect in growth and proliferation with activated T cells capable of completing numerous divisions, though at a reduced rate (Okkenhaug et al., 2002, 2006). Furthermore, T cells expressing a PDK1 K465E mutant which have defective TCR stimulated Akt activity, undergo normal TCR induced growth and proliferation (Waugh et al., 2009). These observations coupled to the fact that T cells activated in limiting concentrations of glucose do show a marked defect in proliferation argue that TCR induced c-Myc expression, glucose uptake and glycolysis is not compromised by disruption of PI3K and Akt signaling (Jacobs et al., 2008). More recently, PI3K/Akt independent glucose uptake and glycolysis in TCR activated T cells has been confirmed using pharmacological inhibitors of PI3Kδ (IC87114) and Akt (Akti1/2) with substantially greater selectivity than LY294002 (Macintyre et al., 2011). Akti1/2 is particularly selective toward Akt due to its unique allosteric mechanism of inhibition, binding to the PH domain of Akt and preventing the PH domain-PI(3,4,5)P_3_ interaction and the resultant conformational change that is a prerequisite for Akt activation (Zhao et al., 2005; Bain et al., 2007). Both IC87114 and Akti1/2 prevent PI3K/Akt signaling in T cells while having no effect on TCR induced glucose uptake (Macintyre et al., 2011). Therefore, it seems clear that PI3K has differential roles in regulating glucose metabolism in developing thymocytes in the thymus and mature T cells in the periphery.

Once activated, T cells differentiate into various different effector T cell subsets depending on the local environment and cytokine availability. Many of these effector T cell subsets maintain an elevated glycolytic rate in response to cytokine signaling (Macintyre et al., 2011; Shi et al., 2011). For example, activated CD8 T cells undergo rapid growth and proliferation in response to interleukin 2 (IL2) as they differentiate into functional CTL. In response to IL2 signaling CTL maintain high levels of glucose uptake and lactate production indicative of elevated glycolysis (Macintyre et al., 2011). IL2 also promotes glucose uptake and glycolysis independently of PI3K and Akt but a key role has been revealed for PDK1. Thus, while IC87114 and Akti1/2 have no effect on CTL glucose uptake, a pronounced decrease is observed following the deletion of PDK1 using a Cre/loxP strategy (Macintyre et al., 2011). While PDK1 is responsible for the activation of Akt, it also activates a number of other members of the AGC kinase family including protein kinase C (PKC), 70-kDa ribosomal S6 kinase (p70S6K), 90-kDa ribosomal S6 kinase (p90RSK), and serum/glucocorticoid regulated kinase (SGK; Pearce et al., 2010). However, unlike the activation of Akt, PDK1 mediated activation of these other AGC family members are independent of PI3K signaling (**Figure 1C**). As members of this kinase family have overlapping substrate specificity it is likely that PDK1 dependent, Akt independent regulation of glucose metabolism reflects functional redundancy within the AGC family of protein kinases (Brunet et al., 2001; Zhang et al., 2006; Sapkota et al., 2007).

mTORC1 has described roles in regulating various aspects of cellular metabolism and given that its activity is independent of PI3K and Akt in some activated T cell subsets, it remains likely that mTORC1 is involved in maintaining glucose uptake and glycolysis (unpublished data; Duvel et al., 2010; Macintyre et al., 2011). Indeed, inhibition of mTORC1 decreases glycolysis in T cells activated under Th17 polarizing conditions (Shi et al., 2011). However, as mTORC1 inhibition also disrupts Th17 differentiation, it is difficult to interpret whether the effect of rapamycin on glycolysis is direct or as a result of the differentiation of different T cells. Therefore, a role for mTORC1 in controlling glycolysis in T cells has still to be formally demonstrated.

## T CELL MIGRATION AND METABOLISM

The expression of adhesion molecules and chemokine receptors orchestrate the peripheral trafficking of activated T cells. The p110γ catalytic subunit is the major PI3K isoform in T cells that promotes PI(3,4,5)P_3_ signaling in response to chemokines. Thus, migration to a range of chemokines is deficient in p110γ^-/-^ T cells and these T cells fail to traffic normally to sites of inflammation (Reif et al., 2004; Smith et al., 2007; Martin et al., 2008; Thomas et al., 2008). T cell migration and motility are energy demanding processes and it is tempting to speculate that chemokine receptor signaling might promote T cell glucose metabolism to meet these energy demands. However, while there is some tentative data linking chemokine receptor signaling to cell growth and metabolism in transformed T cells and developing thymocytes the relationship between chemokine receptor signaling and T cell metabolism has not been directly studied (Janas et al., 2010; Lo et al., 2010). Nonetheless, it is worth mentioning that factors that influence T cell migration and/or homing, and thus the peripheral tissue destination of T cells, will affect T cell metabolism, albeit indirectly, by determining the cytokine environment to which they are exposed. A comparison of activated T cells responding to related cytokines IL2 and IL15 illustrates the differential regulation of T cell metabolism by distinct cytokine environments. IL2 promotes elevated glucose metabolism and glycolysis while IL15 does not maintain this metabolic state and T cells responding to IL15 are smaller with reduced nutrient uptake and glycolysis (Cornish et al., 2006; Macintyre et al., 2011; unpublished data). While PI3Kγ controls T cell migration in response to chemokines, PI3Kδ regulates the repertoire of adhesion and chemokine receptors expressed by activated T cells. PI3Kδ, signaling through Akt and the Foxo transcription factors, regulates the expression of key molecules required for T cell homing between the blood and the lymphoid organs; the adhesion molecule CD62L (also called L-selectin) and the chemokine receptors CC-chemokine receptor 7 (CCR7) and sphingosine-1-phosphate receptor 1 (S1P_1_). Disruption of PI3Kδ/Akt signaling in activated CD8 T cells prevents the down-regulation of CD62L, CCR7, and S1P_1_ and these T cells retain a lymph node trafficking pattern rather than migrating to non-lymphoid tissues and the sites of inflammation (Sinclair et al., 2008; Waugh et al., 2009; Finlay and Cantrell, 2010; Macintyre et al., 2011). Thus, PI3Kδ deficient T cells activated *in vivo* or wild-type T cells activated in mice treated with a PI3Kδ inhibitor fail to traffic into the periphery to antigenic sites (Jarmin et al., 2008). Therefore, both PI3Kγ and δ isoforms coordinately regulate T cell peripheral tissue homing thereby dictating the cytokine environments encountered and indirectly impacting upon T cell metabolism.

## FINAL REMARK

It has recently become clear that the PI3K/Akt signaling axis is not the important regulator of glucose uptake and glycolysis in mature T cells, as initially described. However, disrupting PI3K may in fact impact upon T cell metabolism through indirect mechanisms, i.e., through altering their *in vivo* trafficking pattern, which will dictate the cytokines these T cells encounter.

## Conflict of Interest Statement

The author declares that the research was conducted in the absence of any commercial or financial relationships that could be construed as a potential conflict of interest.
